# Structural, Adsorptive, and Antibacterial Properties of a Novel Silver (Diethyldithiocarbamate)-Decorated Reduced Graphene Oxide Nanocomposite for Sustainable Wastewater Treatment

**DOI:** 10.3390/nano15221709

**Published:** 2025-11-12

**Authors:** Adel Sayari, Hichem Chouayekh, Slim Smaoui, Wajdi Ayadi, Faten M. Ali Zainy, Ahmed S. Badr El-din, Abeer H. Aljadaani, Aida Hmida-Sayari, Amr A. Yakout

**Affiliations:** 1 Department of Biological Sciences, College of Science, University of Jeddah, Jeddah 23890, Saudi Arabia; hschouayekh@uj.edu.sa; 2 Laboratory of Microbial, Enzymatic Biotechnology and Biomolecules, Center of Biotechnology of Sfax, University of Sfax, Sfax 3018, Tunisia; slim.smaoui@cbs.rnrt.tn (S.S.); aida.hmida@cbs.rnrt.tn (A.H.-S.); 3 Laboratory of Molecular Biotechnology of Eukaryotes, Center of Biotechnology of Sfax, University of Sfax, Sfax 3018, Tunisia; wajdi.ayadi.cbs@gmail.com; 4 Chemistry Department, College of Science, University of Jeddah, Jeddah 23890, Saudi Arabia; fmzainy@uj.edu.sa (F.M.A.Z.); ahalgdanee@uj.edu.sa (A.H.A.); 5 Department of Chemistry, Faculty of Science, University of Tabuk, Tabuk 47912, Saudi Arabia; ahmedbadreldin@science.tanta.edu.eg; 6 Chemistry Department, Faculty of Science, Tanta University, Tanta 31527, Egypt; 7 Chemistry Department, Faculty of Science, Alexandria University, Alexandria 5423021, Egypt

**Keywords:** reduced graphene oxide, silver nanocomposite, malachite green adsorption, antibacterial activity, cytotoxicity

## Abstract

Eco-friendly silver nanoparticle systems are highly effective due to their large surface area and strong adsorption capacity. In this study, a novel silver (diethyldithiocarbamate)-decorated reduced graphene oxide nanocomposite (Ag(DDTC)@rGO) was synthesized via a simple green method, yielding a stable and monodispersed material. SEM and HRTEM analyses revealed uniform anchoring of the Ag(DDTC) complex on rGO, producing a coherent nanocomposite with strong physicochemical coupling. The Ag(DDTC)@rGO nanocomposite exhibited a high Brunauer–Emmett–Teller (BET) surface area (289 m^2^ g^−1^) with an average pore diameter of 45 nm, confirming the mesoporous nature of the composite. FTIR spectra showed characteristic bands of rGO and DDTC ligands, with new peaks at 620–640 cm^−1^ confirming the successful anchoring of silver–diethyldithiocarbamate species onto rGO via Ag–S and Ag–O bond formation. Raman spectroscopy further confirmed the multilayered rGO structure after Ag(DDTC) incorporation. X-ray diffraction (XRD) identified a broad hybrid amorphous–crystalline pattern, favorable for catalytic and sensing functions. The superior malachite green adsorption capacity of Ag(DDTC)@rGO was attributed to synergistic electrostatic, π–π stacking, hydrogen bonding, and silver-mediated interactions. Furthermore, antibacterial assays demonstrated significant inhibition of *P. aeruginosa* ATCC 9027 and *S. enterica* ATCC 14028, further enhanced by mild heat activation (40–50 °C) that significantly improved the surface activation of silver nanoparticles. The multifunctional Ag(DDTC)@rGO nanocomposite exhibits strong adsorption and antibacterial properties, highlighting its potential for sustainable wastewater treatment and environmental remediation applications.

## 1. Introduction

The emergence and rapid spread of antibiotic-resistant microorganisms have become a major global health concern, particularly in regions with limited healthcare resources [1,2,3]. Data from the European Antibiotic Resistance Surveillance Network (EARS-Net) indicates that Southern and Eastern Europe have the highest prevalence of resistance, underscoring the urgent need for novel antimicrobial strategies [4]. WHO reports that antibiotic resistance is worsening due to improper antibiotic use [5]. Silver is a leading example among noble metals explored for its strong and well-documented antimicrobial properties [6]. Silver exhibits potent bactericidal activity through multiple mechanisms, including the release of silver ions from particles smaller than 10 nm, direct interaction with bacterial membranes in larger particles (>20 nm), and inhibition of respiratory enzymes via silver–thiol binding [7,8]. However, while silver nanoparticles (AgNPs) possess remarkable antibacterial properties, their potential cytotoxicity toward human cells limits their direct application [9,10]. To mitigate these drawbacks, the development of composite materials incorporating silver has been proposed as a promising approach to balance efficacy and biocompatibility [11].

Several studies have demonstrated that combining silver with other functional materials enhances antimicrobial efficiency. For instance, silver–titanium dioxide nanocomposites synthesized under UV irradiation showed selective antibacterial effects against Gram-negative strains such as *Escherichia coli* and *Klebsiella pneumoniae* compared to Gram-positive species [12]. Similarly, silver-polymer modified graphene oxide composites exhibited temperature-dependent antibacterial activity against *E. coli* and *Staphylococcus aureus* [13]. In addition, silver nanoparticles prepared using green synthesis approaches, such as silk fiber templating, demonstrated not only antibacterial potential but also cytotoxic activity against cancer cells [14]. These findings highlight the versatility of silver-based nanomaterials in addressing microbial resistance and biomedical challenges.

Parallel to their antimicrobial role, nanostructured materials are increasingly investigated for wastewater treatment applications, particularly for the removal of toxic dyes. Conventional adsorbents such as activated carbon, zeolites, mesoporous silica, and metal–organic frameworks have shown limited adsorption capacity and selectivity [15,16,17,18,19,20]. Advanced carbonaceous materials, including fullerenes, nanotubes, and graphite, have also been examined, but their performance remains inadequate for practical dye remediation [21,22,23,24]. In contrast, graphene (G) and graphene oxide (GO) have attracted considerable interest due to their exceptionally high surface area, tunable surface functionalities, and strong π–π and electrostatic interactions, which make them highly effective adsorbents for cationic dyes [25,26,27]. Despite these advantages, many reported GO-based adsorbents suffer from drawbacks such as poor reusability and insufficient mechanistic understanding of dye adsorption [27,28,29].

To address these limitations, in the present study, we report the synthesis of a silver (diethyldithiocarbamate)-decorated reduced graphene oxide nanocomposite (Ag(DDTC)@rGO) and its application for the removal of malachite green (MG) dye from aqueous solutions. Silver-diethyldithiocarbamate complex [Ag(DDTC)] was selected due to its strong metal–sulfur coordination and ability to stabilize silver ions in nanoscale form, which enhances surface reactivity and antimicrobial as well as adsorption properties when supported on reduced graphene oxide. The DDTC ligand provides active sulfur and nitrogen donor atoms that facilitate strong interaction sites for cationic dyes through chelation and surface complexation mechanisms.

The nanocomposite was thoroughly characterized to confirm its successful formation. Batch adsorption experiments were conducted to systematically evaluate the effects of initial dye concentration, pH, contact time, adsorbent dosage, and stirring speed on dye removal. Furthermore, adsorption isotherms, kinetics, reusability, and the underlying adsorption mechanism were investigated to establish the potential of Ag(DDTC)@rGO as a multifunctional nanomaterial with combined antibacterial activity. The cytotoxicity of the nanocomposite was also evaluated.

## 2. Materials and Methods

### 2.1. Reagents

All chemicals used in this study were of analytical reagent grade. Sodium hydroxide (NaOH), silver nitrate (AgNO_3_), hydrochloric acid (HCl), and diethyldithiocarbamate were purchased from Sigma-Aldrich (St. Louis, MO, USA). Malachite green (MG) dye was obtained from Genview Chemical Co. (Houston, TX, USA), while reduced graphene oxide powder was supplied by Sinopharm Inc. Ltd. (Beijing, China). All reagents were used as received without further purification. A stock solution of MG was prepared by dissolving the required quantity of dye in Milli-Q water obtained from a Milli-Q system (Millipore, Billerica, MA, USA), stored at 4 °C, and diluted with fresh Milli-Q water to the desired concentrations prior to each experiment.

### 2.2. Instruments

Pore diameter and surface area were measured using N2 adsorption–desorption analyzer (Nova 4200e, Quantachrome Instruments, Boynton Beach, FL, USA). Spectra were recorded over 190–900 nm; quantification of malachite green was performed at λ*max* = 612 nm, using the UVD-3500 UV-vis spectrophotometer (Labomed Inc., Los Angeles, CA, USA). Ag(DDTC)@rGO nanoparticles’ shape and microstructure were examined using high-resolution field emission transmission electron microscopy (HRTEM, JEOL Ltd., 100 kV, Tokyo, Japan), and scanning electron microscopy (SEM, JEOL JSM-6010LV, Tokyo, Japan). Thermo ESCALAB 250Xi X-ray photoelectron spectroscopy (XPS) (Thermo Fisher Scientific, Waltham, MA, USA) was used to evaluate the valence state and chemical composition of the Ag(DDTC)@rGO nanocomposite. To detect the developed silver nanocomposite, an X-ray powder diffractometer D/MAX-2550 (Rigaku, Tokyo, Japan) equipped with a Cu-Kα radiation source was used. The Ag(DDTC)@rGO nanocomposite’s surface functional groups were characterized by Fourier transform infrared spectrometer (FTIR) with a Nicolet 400 spectrophotometer (Thermo Fisher Scientific, Waltham, MA, USA) using the KBr-pellet method (1 mg sample: 100 mg KBr) at a resolution of 4 cm^−1^ with 32 scans in the 4000–400 cm^−1^ range. Zeta-potential analysis of 1% composite in water was carried out using a Zetasizer nano-Zs90 (Malvern, UK). pH values were determined using a calibrated pH meter (Fischer Scientific, model 810, Pittsburgh, PA, USA).

### 2.3. Green Synthesis of the Ag(DDTC)@rGO Nanocomposite

The Ag(DDTC)@rGO nanocomposite was synthesized via an in situ precipitation method, allowing the silver–diethyldithiocarbamate complex (Ag–DDTC) to form directly in the presence of suspended rGO sheets. This approach promotes intimate contact between the Ag(DDTC) particles and the rGO surface, thereby enhancing the structural stability and dispersion of the active sites. Initially, different mass ratios of rGO were dispersed in 100 mL of deionized water containing 1.0 g of sodium diethyldithiocarbamate (NaDDTC). The suspension was ultrasonicated for 20 min to ensure uniform dispersion of rGO and to facilitate interaction between the rGO sheets and DDTC molecules. Subsequently, 0.1 mol L^−1^ AgNO_3_ solution was added dropwise under continuous stirring. The slow addition promoted controlled nucleation and growth of the Ag(DDTC) complex on the rGO surface. Upon mixing, a yellowish-black precipitate was immediately observed, confirming the successful formation of the nanocomposite. The resulting precipitate was collected via filtration, thoroughly washed with deionized water, followed by ethanol to remove unreacted species and impurities, and then dried in an oven at 60 °C to obtain a stable powder. To further improve homogeneity and reduce particle agglomeration, the dried solid was subjected to mechanical grinding in a ball mill at an oscillation frequency of 25 kHz for 30 min. The final product, Ag(DDTC)@rGO nanocomposite, was obtained as a fine powder suitable for adsorption studies. The Ag(DDTC)@rGO nanocomposite was synthesized via an eco-friendly aqueous route without using any organic solvents or hazardous reducing agents, qualifying as a green synthesis approach consistent with sustainable nanochemistry principles. This synthesis strategy combines the high surface area and π-conjugated framework of rGO with the strong binding affinity of the Ag(DDTC) complex, producing a nanocomposite with enhanced adsorption potential for cationic dyes such as malachite green.

### 2.4. Batch Sorption Study

Batch adsorption experiments were performed to investigate the removal of MG dye by the Ag(DDTC)@rGO nanocomposite under varying experimental conditions. Stock solutions of MG were prepared in Milli-Q water and diluted as required for each trial. The influence of key operational parameters, including initial dye concentration (10–100 mg L^−1^), adsorbent dosage (10–100 mg), solution pH (2–11, adjusted with 0.1 mol L^−1^ NaOH or 0.1 mol L^−1^ HCl), stirring speed (100–400 rpm), and contact time (5–60 min), was systematically evaluated. UV-visible absorption spectra were recorded in the 190–900 nm range using a UVD-3500 spectrophotometer. Quantification of MG concentration was performed by measuring the absorbance at λ_max_ = 612 nm and applying a previously established calibration curve from standard MG solutions. Optimal adsorption conditions were identified as follows: adsorbent dosage of 60 mg, contact time of 40 min, stirring speed of 250 rpm, initial dye concentration of 50 mg L^−1^, and pH ≈ 8.0.

The percentage removal efficiency (%R) was calculated according to Equation (1):

(1) $$\%\;R\;=\frac{C_{0}-C_{e}}{C_{0}}\times 100$$

where *C*_0_ and C_e_ (mg L^−1^) represent the initial and equilibrium dye concentrations, respectively. All experiments were conducted in triplicate, and the reported results correspond to mean values.

### 2.5. Effect of Temperature and Sonication on the Antibacterial Activity of the Nanocomposite

Nine different experimental conditions were used to prepare the nanocomposite samples (Ag(DDTC)@rGO), including different temperatures and sonication times. Using an ultrasonic homogenizer/sonicator (Emmi HC100), Ag(DDTC)@rGO were manufactured by administering the reaction mixtures to ultrasonic waves at 30, 45, and 60 °C for 30, 60, and 90 min at 40 kHz. By employing these experimental conditions, we aimed to investigate the thermal input and sonication time effects on the antibacterial activity of the Ag(DDTC)@rGO. The precise settings for each condition are presented in Table 1.

To assess the antibacterial activity of nanoparticles, food-borne pathogenic bacteria strains were used, including Gram-positive bacteria, *Listeria monocytogenes* ATCC 19117, and Gram-negative bacteria, *Escherichia coli* ATCC 8739, *Salmonella enterica* ATCC 14028, and *Pseudomonas aeruginosa* ATCC 9027. Antibacterial activity was determined using the agar–disk diffusion assay method [30]. Each bacterium was cultured in Luria–Bertani (LB) broth at 37 °C for 24 h and then diluted in Müller–Hinton broth medium at 106 CFU/mL. Each bacterial strain was homogeneously placed on a sterile Petri dish containing Müller–Hinton agar medium. Thereafter, a volume of 100 μL of the Ag(DDTC)@rGO at a concentration of 2 mg mL^−1^ was added to the well positioned on the Petri dish. Incubation of all bacterial plates was carried out for 24 h at 37 °C, followed by measurement of the diameter of the inhibition clear zone in millimeters. The experimental procedure was replicated three times using separate Petri dishes.

### 2.6. MTT Assay

The Ag(DDTC)@rGO submitted to ultrasonic waves at 45 °C for 90 min at 40 kHz (sample 1.6, Table 1) were assessed to determine their cytotoxic effect on Human Umbilical Vein Endothelial Cells (HUVECs). The HUVECs were purchased from a commercial supplier (Gibco, Thermo Fisher Scientific, Waltham, MA, USA). The cells were maintained in Dulbecco’s Modified Eagle Medium (DMEM), supplemented with 10% Fetal Bovine Serum (FBS) and 50 μg/mL gentamicin at 37 °C in a humidified atmosphere containing 5% CO_2_. The cytotoxicity test was performed using a 3-(4,5-dimethyl-2-thiazolyl)−2,5-diphenyl-2H-tetrazolium bromide (MTT) assay as previously reported [31]. Briefly, HUVECs were seeded in a 96-well plate at a density of 5 × 103 cells/well in 100 μL of growth medium and incubated for 24 h at 37 °C under 5% CO_2_ atmosphere. Subsequently, each compound was added to the wells at a final concentration of 25–200 µg/mL. After 24 h incubation, the medium was replaced by fresh medium (100 μL) containing 1 mg mL^−1^ of MTT. After three hours, the formazan product was dissolved in 100 μL DMSO, and the absorbance was measured using a Varioskan Flash microplate reader (Thermo Scientific) at 540 nm. The experiments were conducted in triplicate, and data are expressed as the average of three samples. The IC50 is defined as the concentration of Ag(DDTC)@rGO required to kill 50% of HUVECs.

### 2.7. Statistical Analysis

For Ag(DDTC)@rGO nanoparticle, antibacterial activity experiments were determined in triplicate. A two-way analysis of variance (ANOVA) with two factors (temperature and sonication time as independent factors) was applied by using the Statistical Package for the Social Sciences (SPSS, Version 25, SPSS Ltd., Woking, UK). Means and standard errors were assessed, and a probability level of *p* ≤ 0.05 was utilized in testing the statistical significance of all experimental data. Tukey’s post hoc test was employed to determine the significance of mean values for multiple comparisons at *p* ≤ 0.05.

## 3. Results and Discussion

### 3.1. Characterization and Morphology of the Ag(DDTC)@rGO Nanocomposite

**Figure 1:**
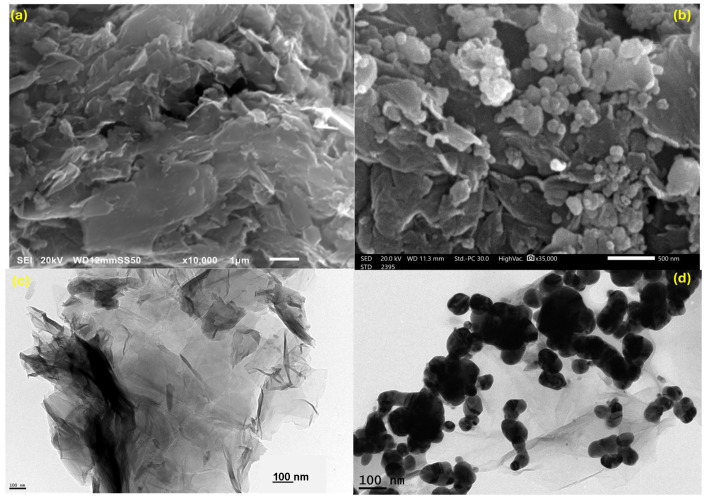
SEM (**a**,**b**) and HRTEM (**c**,**d**) images of G and Ag(DDTC)@rGO nanocomposite.

The SEM and HRTEM micrographs of Ag(diethyldithiocarbamate)@rGOraphene reveal a well-integrated hybrid nanostructure, confirming the successful anchoring of silver-based nanoclusters onto the rGO sheets. The SEM image (Figure 1a) shows the typical wrinkled and layered morphology of reduced graphene oxide, reflecting its high surface area and flexible sheet-like architecture. In contrast, Figure 1b demonstrates the uniform decoration of these sheets with nanosized Ag(diethyldithiocarbamate) particles, which appear as bright, spherical entities densely distributed across the carbon framework, suggesting strong interfacial interactions between the metal complex and the reduced graphene oxide surface. The HRTEM images (Figure 1c,d) further substantiate this observation, where the transparent, thin rGO layers can be seen providing a support matrix for the well-dispersed, dark contrast Ag nanoparticles. The particles display near-spherical morphology with nanoscale dimensions, indicating limited aggregation and good stabilization by the diethyldithiocarbamate ligands. The intimate contact between rGO and the Ag complex points to efficient electron delocalization and interfacial charge transfer, a feature that enhances the potential catalytic and electronic behavior of the composite. Overall, the combined SEM-HRTEM analysis confirms that the Ag(diethyldithiocarbamate) complex is homogeneously anchored on rGO, forming a structurally coherent nanocomposite with both high dispersion and strong physicochemical coupling.

Nitrogen adsorption–desorption isotherms demonstrated type-IV curves with a clear H3 hysteresis loop, characteristic of mesoporous materials. The Brunauer–Emmett–Teller (BET) surface area of Ag(DDTC)@rGO was measured to be 289 m^2^ g^−1^, slightly higher than pristine rGO (247 m^2^ g^−1^), due to the intercalation of Ag nanoparticles preventing sheet restacking. The Barrett–Joyner–Halenda (BJH) pore size distribution indicated average pore diameters of ~45 nm, confirming the mesoporous nature of the composite. The increased surface area and uniform porosity are advantageous for dye adsorption, providing abundant active sites and facilitating efficient mass transfer.

**Figure 2:**
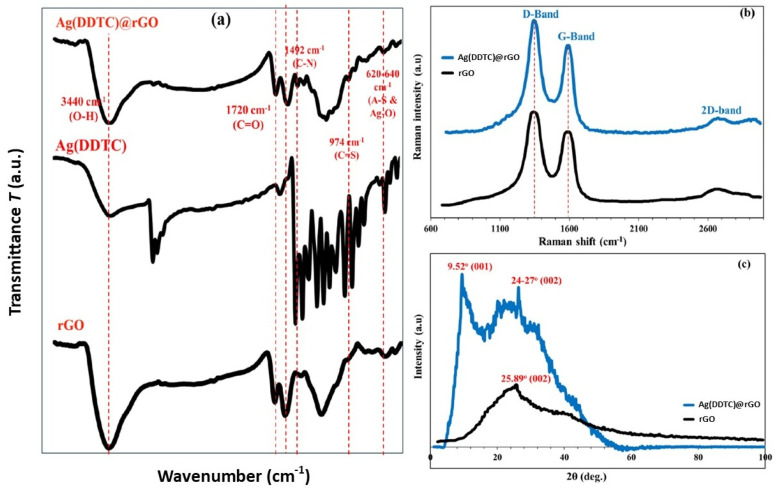
FTIR (**a**), Raman (**b**), and XRD (**c**) results of the Ag(DDTC)@rGO nanocomposite.

The FTIR spectrum of the Ag(DDTC)@rGO nanocomposite (Figure 2a) exhibited distinct vibrational bands associated with both rGO and DDTC ligands. A broad band around 3400–3420 cm^−1^ corresponded to –OH stretching vibrations, indicating residual hydroxyl functionalities on the rGO surface [32]. The band observed near 1722 cm^−1^ was assigned to the C=O stretching of carboxyl groups, while the peak at 1598 cm^−1^ corresponded to C=C skeletal vibrations of the sp^2^-hybridized rGO framework. The strong broad –OH stretching band (≈3420 cm^−1^) and C=O stretching at 1722 cm^−1^ indicate that the carbon framework retains abundant hydroxyl and carbonyl groups, confirming a partially reduced graphene oxide (rGO) structure rather than completely reduced graphene. In addition, characteristic bands of DDTC were detected, including C–N stretching at 1492 cm^−1^ and C=S stretching vibrations at 974 cm^−1^, confirming the presence of the Ag-DDTC complex [33,34]. A new band appearing around 620–640 cm^−1^ was attributed to Ag–S and Ag–O vibrations, evidencing the successful anchoring of silver–diethyldithiocarbamate species onto reduced graphene oxide [35].

Raman spectroscopy (Figure 2b) further confirmed the structural modifications of rGO after Ag(DDTC) incorporation. Two prominent bands were observed: the D band (~1342 cm^−1^), arising from structural defects and disorder in the rGO lattice, and the G band (~1595 cm^−1^), corresponding to the in-plane vibrations of sp^2^ carbon atoms.

The intensity ratio (*I_D_*/*I_G_*) increased from 0.83 for pristine reduced graphene oxide to 0.97 for Ag(DDTC)@rGO, indicating an increase in defect density due to the interaction of DDTC and Ag species with the rGO surface [36,37]. This modification is consistent with successful functionalization and nanoparticle decoration. Additionally, the appearance of weak bands in the 2700 cm^−1^ region (2D band) confirmed the multilayered rGO structure, while slight shifts in the G band indicated charge transfer interactions between rGO and the Ag(DDTC) complex.

The XRD pattern of Ag(diethyldithiocarbamate)@rGO (Figure 2c) exhibits a broad and diffused diffraction profile, characteristic of materials with low crystallinity or partially amorphous structures. The diffraction pattern of pure reduced graphene oxide typically shows a sharp reflection at 2θ = 9.52°, indexed to the (001) plane [38]. The broad hump observed around 2θ ≈ 24–27° corresponds to the (002) reflection of graphitic carbon, confirming the presence of few-layer or disordered rGO sheets [39]. The diffraction peak for the native rGO appeared at 2θ = 25.89°, corresponding to the (002) basal plane. The absence of sharp, intense peaks suggests that the rGO support remains largely exfoliated and structurally defective, favoring high surface area and active sites for metal anchoring. The small, poorly resolved features appearing in the 2θ range of 35–45° may be associated with the formation of finely dispersed silver nanodomains within the rGO matrix, corresponding to the (111) and (200) planes of face-centered cubic (FCC) silver [40]. This broadness reflects nanoscale crystallite formation and strong metal–support interactions. The overall pattern implies that the Ag(diethyldithiocarbamate) complex is successfully immobilized on rGO without causing significant graphitic restacking or crystallite growth, highlighting the composite’s hybrid amorphous/crystalline nature, an advantageous structural feature for catalytic, electronic, or sensing applications.

### 3.2. Batch Sorption Parameters

#### 3.2.1. Effect of Initial Dye Concentration

**Figure 3:**
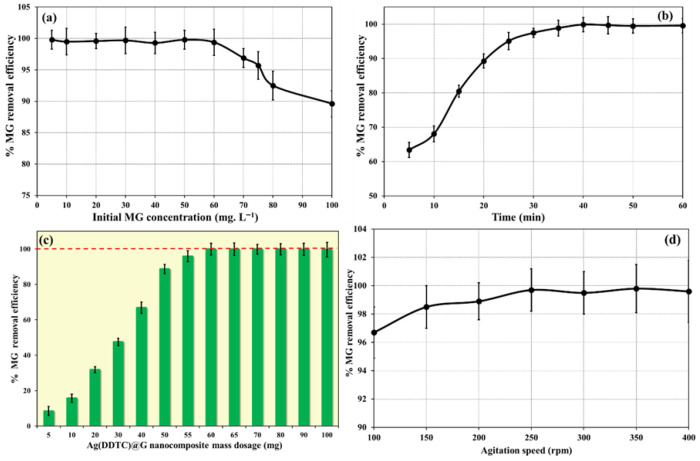
Impact of initial MG concentration (**a**), time (**b**), mass dosage (**c**), and agitation speed (**d**) on MG removal efficiency.

The influence of the initial MG concentration on adsorption performance is illustrated in Figure 3a. Batch experiments were conducted with MG concentrations ranging from 10 to 100 mg L^−1^, while maintaining constant conditions of stirring speed of 250 rpm, 40 min contact time, temperature of 25 °C, 60 mg nanosorbent dosage, and pH of 8.0. The removal efficiency remained nearly constant up to 50 mg L^−1^, beyond which a decline was observed. This reduction is attributed to the progressive saturation of available surface binding sites as the dye concentration increases, thereby limiting further adsorption. Comparable concentration-dependent trends have been documented in previous studies [41,42,43].

#### 3.2.2. Effect of Contact Time

The effect of contact time on MG removal is presented in Figure 3b. Experiments were performed with varying contact times (5–60 min) under fixed conditions: adsorbent dosage, 60 mg; stirring speed, 250 rpm; MG concentration, 50 mg L^−1^; and pH, 8.0. The removal efficiency increased rapidly with contact time due to more frequent interactions between MG molecules and active sites of the adsorbent. Equilibrium was reached at approximately 40 min, after which no significant improvement was observed, indicating saturation of binding sites on Ag(DDTC)@rGO. This equilibrium behavior is consistent with earlier findings [42,43,44].

#### 3.2.3. Effect of Ag(DDTC)@rGO Mass Dosage

The effect of Ag(DDTC)@rGO dosage on MG adsorption efficiency is shown in Figure 3c. Dosages ranging from 5 to 100 mg were tested, while other parameters (25 °C, pH 8.0, MG concentration 50 mg L^−1^, stirring speed 250 rpm, and contact time 40 min) were kept constant. Removal efficiency improved with increasing dosage due to the availability of additional binding sites. Maximum efficiency was achieved at 60 mg, beyond which further increases in dosage produced no significant change, suggesting that most dye molecules had already been adsorbed. Similar saturation trends have been reported previously [36,43,45].

#### 3.2.4. Effect of Stirring Speed

The influence of stirring speed on adsorption efficiency is depicted in Figure 3d. Experiments were conducted at stirring speeds ranging from 100 to 400 rpm, fixing all other parameters (pH: 8.0, mass dosage: 60 mg, contact time: 40 min). The removal efficiency increased with the stirring speed up to 250 rpm, likely due to enhanced mass transfer and increased collision frequency between dye molecules and adsorbent particles. Beyond this point, efficiency remained constant, indicating that adsorption had reached its maximum potential. Similar behavior has been reported by other researchers [44].

#### 3.2.5. Effect of pH

**Figure 4:**
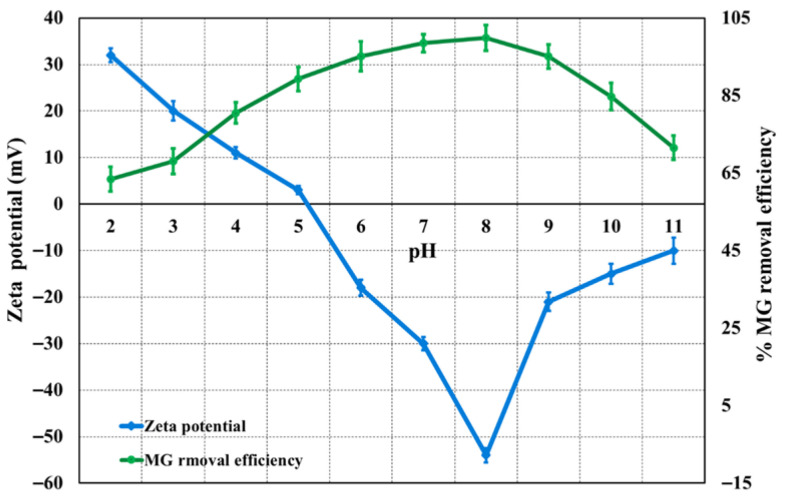
Impact of pH on MG removal efficiency (optimum conditions: 50 mg L^−1^ of MG, 60 mg, 250 rpm, and 40 min contact time).

The influence of solution pH on MG removal by Ag(DDTC)@rGO is presented in Figure 4. Adsorption experiments were conducted over a pH range of 2–11, with fixed conditions of adsorbent dosage (60 mg), stirring speed (250 rpm), and contact time (40 min). The pH of the solutions was adjusted using 0.1 mol L^−1^ NaOH or 0.1 mol L^−1^ HCl. As shown in Figure 4, the MG removal efficiency increased steadily from pH 2 to 8, beyond which it remained nearly constant. This behavior can be explained by the electrostatic interactions between the cationic MG dye and the negatively charged surface of Ag(DDTC)@rGO, as confirmed through the zeta potential analysis (Figure 4). The nanocomposite exhibited a zeta potential of −53.6 mV (pH 8), indicating good colloidal stability and a negatively charged surface favorable for electrostatic interaction with positively charged malachite green molecules during adsorption. At lower pH values, the weaker electrostatic attraction reduced removal efficiency, whereas at pH 8, strong electrostatic interactions facilitated maximum adsorption. Further increases in pH did not enhance adsorption, suggesting that optimum performance was achieved at pH 8. Consequently, this pH was selected for subsequent experiments.

### 3.3. Kinetic Study of Adsorption

Kinetic analysis is fundamental to understanding the adsorption behavior of dyes, as it provides insights into the rate-limiting steps, diffusion pathways, and overall mechanism governing the process. It also serves as a practical tool for designing and optimizing large-scale adsorption systems [46,47]. To investigate the adsorption mechanism of MG dye onto the Ag(DDTC)@rGO nanocomposite, experimental data were fitted to three widely applied kinetic models: the pseudo-first order (PFO), pseudo-second order (PSO), and intra-particle diffusion (IPD) models.


(2) $$\frac{t}{q_{t}}=\frac{1}{k_{2}q_{e}^{2}}+\frac{t}{q_{e}}\;\;\;\;\;\mathrm{PSO}$$

(3) $$\ln\;\left(q_{e}-q_{t}\right){=\ln\;q}_{e}-k_{1}t\;\;\;\;\;\mathrm{PFO}$$

(4) $$q_{t}=K_{p}\;t^{0.5}+c\;\;\;\;\;\mathrm{IPD}$$

**Figure 5:**
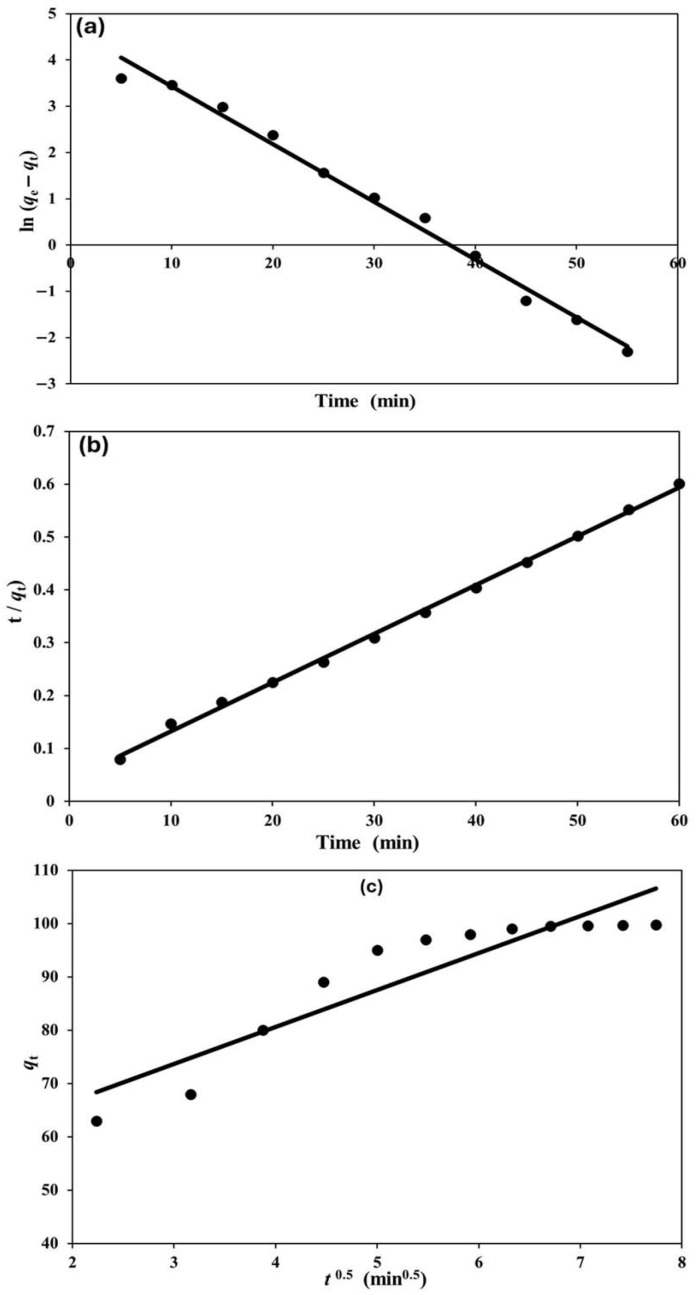
Evaluation of kinetic behavior for MG adsorption onto Ag(DDTC)@rGO using PFO (**a**), PSO (**b**), and IPD (**c**) models, respectively.

The kinetic behavior of MG adsorption on the Ag(DDTC)@rGO nanocomposite by the three models was evaluated and displayed, as shown in Figure 5. The linearized form of the PFO model (Equation (2)) assumes that the rate of sorption is directly proportional to the number of unoccupied active sites. From the slope and intercept of the plot of log(*q_e_ − q_t_*) versus time, the equilibrium adsorption capacity (*q_e_*), rate constant (*K*_1_), and correlation coefficient (R^2^) were calculated. Although this model provided a reasonable fit, the correlation values (*R^2^* ≈ 0.9895) indicated that it did not fully capture the adsorption behavior.

The PSO model (Equation (3)) describes adsorption based on the assumption that chemisorption, involving electron sharing or exchange between adsorbate and adsorbent, is the rate-determining step. A linear relationship was observed when plotting *t*/*qt* against t, and the calculated parameters from the linear form of the model demonstrated excellent agreement between experimental and theoretical q_e_ values. The high correlation coefficient (*R*^2^ ≈ 0.9980) confirmed that MG adsorption on Ag(DDTC)@rGO is best represented by the PSO model, supporting the dominance of chemisorption processes [48,49].

To further explore mass transfer effects, the intra-particle diffusion model (Equation (4)) was applied. The plot of *qt* versus *t*^0.5^ exhibited multilinearity, suggesting that more than one step governs the overall adsorption. The intraparticle diffusion fit is not linear through the origin (*R*^2^ ≈ 0.8478, C ≈ 44 mg g^−1^), meaning that pore diffusion contributes but is not the sole rate-limiting step; boundary-layer (film) resistance is present at early times. The initial sharper region corresponds to boundary layer (film) diffusion; in comparison, the subsequent linear portion is attributed to intra-particle diffusion within the porous structure of the nanocomposite. Since the lines did not pass through the origin, it can be inferred that intra-particle diffusion was not the sole rate-limiting step; instead, both external mass transfer and intra-particle diffusion jointly controlled the process [50].

Overall, the kinetic evaluation (Table 2) revealed that MG dye adsorption on Ag(DDTC)@rGO follows a pseudo-second-order mechanism, highlighting the importance of chemical interactions such as electrostatic attraction and π–π stacking. Moreover, the contribution of diffusion resistance, as indicated by the intra-particle model, demonstrates the role of both surface accessibility and pore structure in adsorption efficiency. These findings confirm that the adsorption of MG dye on Ag(DDTC)@rGO is governed by a synergistic mechanism involving chemisorption and diffusion processes, consistent with previous reports on dye–nanocomposite systems [51,52].

### 3.4. Regeneration and Reusability of Ag(DDTC)@rGO

**Figure 6:**
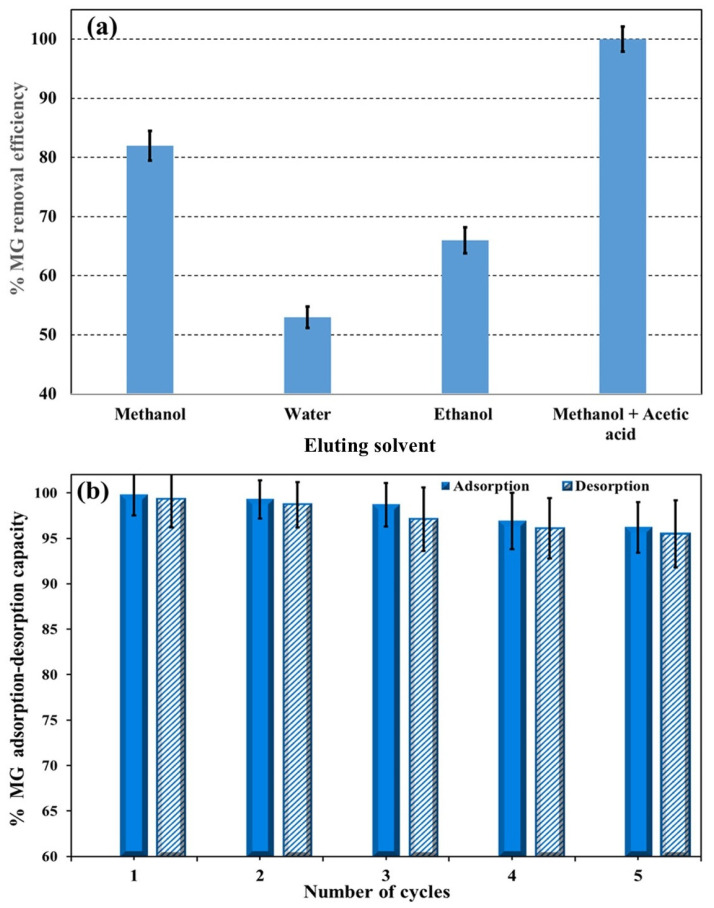
(**a**) Reusability of the Ag(DDTC)@rGO nanocomposite using water, methanol, ethanol, and methanol + 5% acetic acid (*v*/*v*). (**b**) Desorption–adsorption cycles using methanol + 5% acetic acid (*v*/*v*).

Reusability is a key parameter in evaluating the economic feasibility and environmental sustainability of an adsorbent. To assess the MG removal efficiency of Ag(DDTC)@rGO, several reagents, including Milli-Q water, ethanol, methanol, and methanol + 5% acetic acid (*v*/*v*), were tested. Among these methods, MG extraction/elution, which reports top recoveries with methanol + 5% acetic acid (*v*/*v*), proved to be the most effective desorbing agent (Figure 6a). After each adsorption cycle, the dye-loaded nanosorbent was treated with the methanol/acetic acid mixture, washed, oven-dried, and reused in subsequent adsorption experiments. The regenerated nanosorbent maintained high efficiency over multiple cycles, with the MG dye removal efficiency decreasing to 95.5% after five successive reuses (Figure 6b). These results demonstrate the strong reusability and stability of the Ag(DDTC)@rGO nanocomposite, highlighting its potential for practical wastewater treatment applications. Furthermore, the reported adsorption capacities of various graphene-based and metal-modified nanocomposites for MG removal demonstrated that the performance of Ag(DDTC)@rGO (≈91 mg g^−1^) is comparable or superior to values reported for other graphene-based and metal-modified adsorbents, such as GO [53,54], ZnO/G [55,56], and Ag-decorated carbon materials [57,58], which typically exhibit capacities in the range of 30–90 mg g^−1^. The Ag(DDTC)@rGO nanocomposite achieved comparable or higher efficiency under mild conditions, confirming its competitive sorption potential.

### 3.5. Mechanism of MG Dye Adsorption on the Ag(DDTC)@rGO Nanocomposite

The mechanism of malachite green adsorption onto Ag(DDTC)@rGO can be attributed to a combination of physicochemical interactions, including hydrogen bonding, electrostatic attraction, π–π stacking, and non-covalent forces. Control adsorption tests using pristine reduced graphene oxide under identical conditions (pH 8, 25 °C, C_0_ = 50 mg L^−1^) revealed a lower capacity (53 mg g^−1^) compared with Ag(DDTC)@G (91 mg g^−1^), confirming the synergistic enhancement arising from Ag-mediated surface activation and increased electron density. At pH 8, the surface of Ag(DDTC)@rGO exhibits a negative charge, as confirmed through zeta potential analysis. This negative surface charge originates from the sp^2^-hybridized carbon frameworks of graphene and its π-aromatic structure, which facilitates electrostatic attraction with the cationic MG molecules. The dimethylamine functional groups of MG enhance this interaction, resulting in strong binding between the dye and the nanocomposite surface [35,56,57,58,59,60,61]. π–π stacking interactions between the aromatic rings of MG and the conjugated graphene domains further contribute to adsorption, promoting dye immobilization within the porous nanocomposite structure [62,63]. The embedded silver (diethyldithiocarbamate) nanoparticles introduce additional active sites that participate in weak non-covalent forces and coordination-type interactions with MG molecules. These interactions not only increase the adsorption capacity but may also contribute to electron transfer processes, enhancing dye–adsorbent affinity [64,65]. Overall, the synergistic contribution of electrostatic interactions, π–π stacking, hydrogen bonding, and silver-mediated non-covalent forces explains the high efficiency of MG removal by Ag(DDTC)@rGO. Such a multi-mechanistic adsorption pathway has also been reported for other dye–nanocomposite systems, reinforcing the reliability of this mechanism [65,66,67].

### 3.6. Antibacterial Activity and Cytotoxicity

#### 3.6.1. Impact of Temperature and Sonication on Ag(DDTC)@rGO’ Antibacterial Activity

In this section of the paper, we intend to assess the influence of temperature and sonication time on the antibacterial activity, expressed in mm, of the Ag(DDTC)@rGO. Under nine different experimental conditions, combining three temperatures (30 °C, 45 °C, and 60 °C) with three sonication times (30, 60, and 90 min), the antibacterial activity was assessed against four bacterial strains, viz. *L. monocytogenes*, *S. enterica*, *E. coli*, and *P. aeruginosa* (Table 3). Under controlled temperature conditions, the antibacterial activity as a function of sonication time was evaluated. In this line, at 30 °C, the prolonging of the sonication time resulted in a moderate (*p* ≤ 0.05) but reliable increase in the inhibition zones for most target microorganisms. For instance, the antibacterial effect on *S. enterica* increased from 12 mm (30 min) to 16 mm (90 min); in comparison, that of *P. aeruginosa* increased from 15 mm to 16 mm (Table 3). In addition, a slight increase (*p* ≤ 0.05) in *E. coli* inhibition was observed. These results reveal that longer ultrasonic treatment improves the dispersal and surface activation of nanoparticles, possibly helping the interactions with bacterial cell membranes.

Comparable trends were detected at 45 °C, with *L. monocytogenes* displaying a significant increase (*p* ≤ 0.05): from 9 mm (30 min) to 14 mm (90 min). For other strains, the inhibitory effects were less marked. In contrast, at 60 °C, the sonication time was less appreciable (*p* ≤ 0.05), illustrating higher temperatures may reduce the benefits of long sonication, possibly due to the agglomeration or degradation of active agents [68]. In other studies, similar results have shown that sonication plays a vital role in breaking down nanoparticle aggregates, triggering improvements in dispersion and homogeneity in colloidal suspensions. The authors of such studies have reported that deagglomeration increases the nanoparticle surface area and therefore enhances antimicrobial potential [68]. Likewise, other studies have highlighted the impact of ultrasonication in controlling the physicochemical properties of metallic nanoparticles [69]. The authors of such studies note that the mechanical energy transferred by ultrasonic waves can disperse nanoparticles more consistently and induce surface activation by generating defects and roughening surfaces. These changes could improve the chemical reactivity of nanoparticles, aiding resilient interactions with bacterial membranes and augmenting their antimicrobial potential.

Moreover, at a fixed sonication time, the temperature could mark the antibacterial activity by modifying nanoparticle performance and efficacy. In this respect, when examining the impact of temperature alone, a non-linear response was perceived (Table 3). For example, after 30 min of sonication, the increase in temperature from 30 °C to 60 °C could significantly (*p* ≤ 0.05) reduce *P. aeruginosa* growth. Alternatively, *E. coli* persists moderately unaffected (*p* > 0.05) (12 mm across all temperatures).

At 90 min of sonication time, the greatest inhibition diameter against *L. monocytogenes* was found at 45 °C (14.83 mm), exceeding findings at 30 °C (9 mm) and 60 °C (10.25 mm). This finding indicates that intermediate temperature heat (45 °C) may endorse nanoparticle interaction with Gram-positive bacterial membranes without affecting thermal denaturation of the active agent. These results are in line with other findings reporting that heat treatment in the range of 40–50 °C significantly improved the surface activation of silver nanoparticles, engendering an increase in the inhibition of *S. aureus* and *E. coli* [70]. Nevertheless, when the temperature surpassed 60 °C, a distinct decline in efficacy was detected [70]. Equally, other authors demonstrated that the antibacterial activity of ZnO nanoparticles decreased by more than 40% when a temperature shift from 60 °C to 80 °C was applied during treatment [71]. These outcomes indicate that moderate temperature could improve nanoparticle function, while extreme temperatures damage their structure and antimicrobial activity.

Between the four tested strains, *P. aeruginosa* ATCC 9027 and *S. enterica* ATCC 14028 showed the maximum inhibition zones, achieving zones of 16 mm; however, under all treatment conditions, *E. coli* ATCC 8739 remained stable (12–13 mm). *L. monocytogenes* ATCC 19117, a Gram-positive bacterium, presented higher sensitivity to changes in sonication time and temperature, representing a different interaction mechanism associated with cell wall structure. This variable response might be credited to the structural differences between Gram-negative and Gram-positive bacteria. The studied Gram-negative strains (*E. coli* ATCC 8739, *S. enterica* ATCC 14028, and *P. aeruginosa* ATCC 9027) possess an outer membrane that could obstruct nanoparticle diffusion, whereas Gram-positive strains possess a denser peptidoglycan layer, which may permit more direct interactions. Overall, the results indicate that prolonged sonication could enhance antibacterial activity by augmenting compound dispersion and stability, whereas modest temperature (≤40 °C) induces microbial interaction without affecting degradation.

#### 3.6.2. Cell Viability

**Figure 7:**
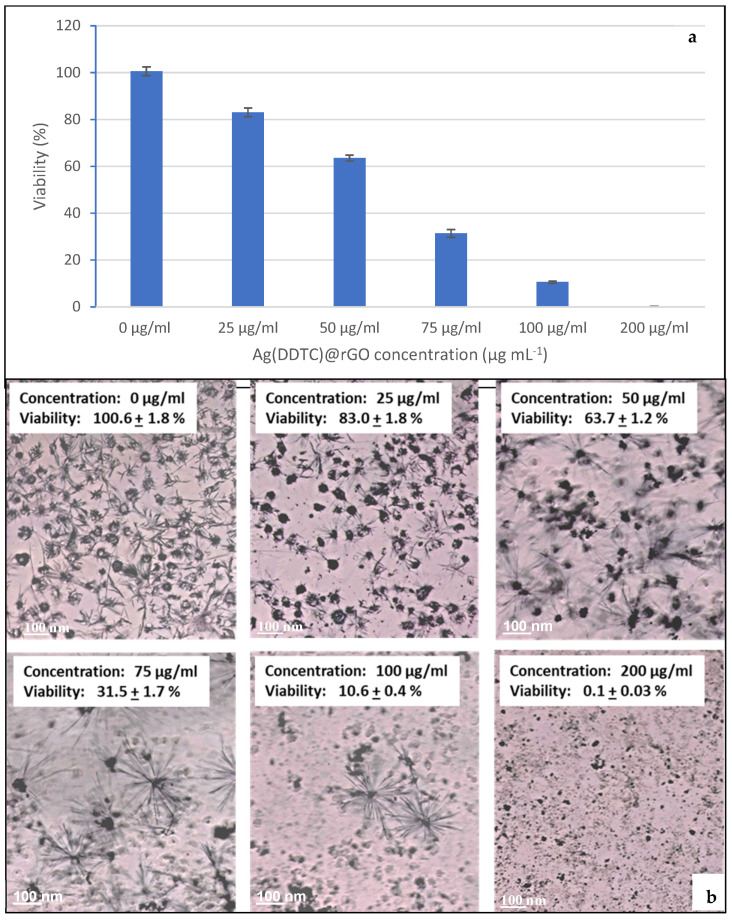
Effect of different concentrations of the Ag(DDTC)@rGO nanocomposite on the cell viability of HUVECs (**a**). The corresponding micrographs (**b**) with scale bar = 100 μm. The Ag(DDTC)@rGO were submitted to ultrasonic waves at 45 °C for 90 min at 40 kHz (sample 1.6, Table 1). Cytotoxicity was measured, using the MTT assay, after exposing HUVECs to various concentrations of Ag(DDTC)@rGO (25, 50, 75, 100, and 200 µg/mL).

The cytotoxic effect of the Ag(DDTC)@rGO submitted to ultrasonic waves at 45 °C for 90 min at 40 kHz was evaluated on HUVECs using an MTT colorimetric assay. Cells were exposed to various concentrations of Ag(DDTC)@rGO (25, 50, 75, 100, and 200 µg/mL) for 24 h. Untreated cells served as the control group, with cell viability defined as 100%. As shown in Figure 7, cell viability decreased progressively with increasing Ag(DDTC)@rGO concentrations starting from 25 µg/mL, indicating a dose-dependent cytotoxic effect (Figure 7a,b). The Ag(DDTC)@rGO presented an IC50 of 64.11 µg/mL with a complete cytotoxic effect at a concentration of 200 µg/mL.

The observed IC_50_ of 64 µg/mL is in line with reported works. Indeed, IC_50_ values of 10.7 to 75.9 µg/mL were reported for NM-300K silver nanoparticles across fish cell lines after 24 h exposure [72]. Similarly, IC_50_ values of 25 µg/mL and 70 µg/mL were reported for biologically and chemically synthesized Ag(DDTC)@rGO, respectively, in human cell systems [73]. The higher IC_50_ in our case may reflect the stabilizing effect of the composite matrix, restricted Ag^+^ release, or steric hindrance limiting cellular uptake.

## 4. Conclusions

The Ag(DDTC)@rGO nanocomposite was successfully synthesized via a green approach, exhibiting uniform structural integration, high surface area, and mesoporosity, enabling efficient adsorption and antibacterial activity. Malachite green removal power followed pseudo-second-order kinetics, indicating chemisorption governed by electrostatic and π–π interactions. The nanocomposite maintained high reusability with minimal loss in performance. Enhanced antibacterial efficacy, particularly under moderate heat and sonication, confirmed its multifunctional potential. Overall, Ag(DDTC)@rGO represents a sustainable, reusable, and biocompatible nanomaterial for wastewater treatment and microbial control.

## Figures and Tables

**Table 1:** Combination of temperature and sonication time used in the preparation of the Ag(DDTC)@rGO sample.

Condition	Temperature (°C)	Sonication Time (min)
1.1	30	30
1.2	30	60
1.3	30	90
1.4	45	30
1.5	45	60
1.6	45	90
1.7	60	30
1.8	60	60
1.9	60	90

**Table 2:** Kinetic parameters for the different adsorption models for MG adsorption onto the Ag(DDTC)@rGO nanocomposite (conditions: 25 °C, pH 8, C_0_ = 50 mg L^−1^, adsorbent = 60 mg, stirring = 250 rpm).

Kinetic Model	Kinetic Parameters		R^2^
	*q*_e,calc._ (mg g^−1^)	91.5	
PFO ^1^	*q*_e,exp._ (mg g^−1^)	81.9	0.91
	*K*_1_ (min^−1^)	0.144	
PSO ^1^	*q*_e,exp._ (mg g^−1^)	90.8	0.993
	*K*_2_ (g mg^−1^ min^−1^)	4.47 × 10^−3^	
IPD ^1^	*K*_p_ (mg g^−1^ min^−1/2^)	5.73	0.898
	*C*	44.4	

^1^ PFO: Pseudo-first order; PSO: Pseudo-second order; IPD: Intra-particle diffusion. *K*_1_, *K*_2_, and *K_p_*: Rate constants for PFO, PSO, and intra-particle (IP) models. *q_e_*: calculated (*q_e_* calc.) and experimental (*q_e_* exp.) equilibrium adsorption capacity (mg g^−1^). *q_t_*: adsorption capacity at time t (mg g^−1^) h.

**Table 3:** Antibacterial activity, expressed in mm, of Ag(DDTC)@rGO under different temperature and sonication conditions.

Sample	1.1	1.2	1.3	1.4	1.5	1.6	1.7	1.8	1.9
*L. monocyogenes* ATCC 19117 (mm)	11.75 ± 0.52 ^aAB^	12.00 ± 0.55 ^aB^	12.00 ± 0.5 ^aB^	12.00 ± 0.55 ^aB^	12.00 ± 0.5 ^aB^	12.00 ± 0.55 ^aB^	12.00 ± 0.5 ^aB^	12.00 ± 0.55 ^aB^	12.00 ± 0.5 ^aB^
*S. enterica *ATCC 14028 (mm)	12.33 ± 0.59 ^aAB^	15.0 ± 0.71 ^bC^	16.66 ± 0.78 ^cD^	15.0 ± 0.71 ^bC^	16.66 ± 0.78 ^cD^	15.0 ± 0.71 ^bC^	16.66 ± 0.78 ^cD^	15.0 ± 0.71 ^bC^	16.66 ± 0.78 ^cD^
*E. coli *ATCC 8739 (mm)	12.50 ± 0.62 ^aA^	12.33 ± 0.57 ^aA^	13.83 ± 0.65 ^bA^	12.33 ± 0.57 ^aA^	13.83 ± 0.65 ^bA^	12.33 ± 0.57 ^aA^	13.83 ± 0.65 ^bA^	12.33 ± 0.57 ^aA^	13.83 ± 0.65 ^bA^
*P. aeruginosa *ATCC 9027 (mm)	15.83 ± 0.7 ^bC^	15.75 ± 0.72 ^bC^	16.75 ± 0.75 ^cD^	15.75 ± 0.72 ^bC^	16.75 ± 0.75 ^cD^	15.75 ± 0.72 ^bC^	16.75 ± 0.75 ^cD^	15.75 ± 0.72 ^bC^	16.75 ± 0.75 ^cD^

Samples 1.1–1.9 correspond to different combinations of temperature (30 °C, 45 °C, and 60 °C) and sonication time (30, 60, 90 min) as described in Table 1. Superscripts within columns (a, b, c) indicate significant differences (*p* ≤ 0.05) between the four target microorganisms for the same treatment. Superscripts within rows (A, B, C, D) indicate significant differences (*p* ≤ 0.05) between the treatment conditions for the same bacterial group. Values are expressed as the mean ± standard deviation (mm) of inhibition zones.

## Data Availability

The original contributions presented in this study are included in the article. Further inquiries can be directed to the corresponding authors.
